# Fabrication and Application of Tannin Double Quaternary Ammonium Salt/Polyvinyl Alcohol as Efficient Sterilization and Preservation Material for Food Packaging

**DOI:** 10.3390/molecules29174264

**Published:** 2024-09-09

**Authors:** Laiqi Li, Wenke Zha, Ximei Huang, Yangyi Gong, Sufang Li

**Affiliations:** 1 Solid-State Ion Institute, College of Chemistry and Chemical Engineering, Hu-Nan University, Changsha 410006, China; laiqili@hnu.edu.cn (L.L.); science@hnu.edu.cn (Y.G.); 2 Advanced Energy Storage Key Technology and Reliability R&D Center (AESC), School of Mechanical Engineering, Anhui Science and Technology University, Chuzhou 233100, China; 3 Zhongshan Nenghe Biotech Co., Ltd., Zhongshan 528400, China; ximei88huang@hotmail.com

**Keywords:** tannic acid, bisquaternary ammonium salt, water-based packaging material, antibacterial, antioxidant

## Abstract

Food packaging films play a vital role in preserving and protecting food. The focus has gradually shifted to safety and sustainability in the preparation of functional food packaging materials. In this study, a bisquaternary ammonium salt of tannic acid (BQTA) was synthesized, and the bioplastics based on BQTA and polyvinyl alcohol (PVA) were created for packaging applications. The impact of BQTA on antibacterial effect, antioxidant capacity, opacity, ultraviolet (UV) protective activity, mechanical strength, thermal stability, and anti-fog of the resultant bioplastics was examined. In vitro antibacterial experiments confirmed that BQTA possesses excellent antimicrobial properties, and only a trace amount addition of BQTA in PVA composite film could inhibit about 100% of *Escherichia coli* and *Staphylococcus aureus*. Compared to BQTA/PVA bioplastics with pure PVA, the experiment findings demonstrate that BQTA/PVA bioplastics show strong antioxidant and UV protection action and the performance of fruit preservation. It also revealed a small improvement in thermal stability and tensile strength. The small water contact angle, even at low BQTA concentrations, gave BQTA/PVA bioplastics good anti-fog performance. Based on the findings, bioplastics of BQTA/PVA have the potential to be used to create packaging, and they can be applied as the second (inner) layer of the primary packaging to protect food freshness and nutrition due to their antioxidant activity and biocompatibility.

## 1. Introduction

Food spoilage caused by foodborne pathogens and microorganisms is a serious matter [1]. The traditional preservation strategy inhibiting microbial growth is the direct usage of preservatives, such as sorbate, nitrite, benzoate, and so on, which have negative impacts on fresh taste, texture, and nutritional composition of fruits, vegetables, and meats. The preferred method of improving the shelf life of the food is exploring and developing food packaging material, which generally displays excellent features of antibacterial, antioxidation, water vapor transmission, and biocompatibility [2]. Modern food packaging materials are mainly composed of biodegradable polymer matrix [3] and some active additives in the matrix. The active additives are commonly referred to as synthetic inorganic nanoparticles, such as nano-Ag [4], cobalt-citrate [5], ZnO [6], TiO_2_ [7], carbon dots [8] and synthetic antimicrobial peptides [9], enzyme [10], besides, some natural polyphenols biomolecules extracted from plants [11,12,13,14,15]. The antibacterial effect of natural polyphenols is not obvious, and the flavor or odor is unwelcome. Uneven distribution of inorganic nanoparticles in the matrix suppressed their antibacterial effect in food packaging. A hot spot of food packaging is to research low-toxicity and efficient antimicrobials with flavorless, odorless, and highly selective permeable qualities to maintain the sensory and nutritional properties of food during storage.

It is well known that quaternary ammonium salts (QASs) are strong antibacterial compounds that can disrupt bacterial normal structure by the reaction between positively charged N atoms of amino groups and the negatively charged cell walls of bacteria [16]. The literature indicates that QASs with 12 carbon chains show the highest biocidal activity, while the extension and the reduction in the length of the alkyl chain weaken the antimicrobial activity [17]. Furthermore, the antibacterial activity of QASs is closely related to the number of ammonium fragments. The higher the charge density in the presence of more ammonium segments, the stronger the action on the membrane of bacteria [18,19,20,21]. Nevertheless, frontier reports have found that QASs are also lethal to normal cells [16]. The ways of using QASs are physical blends and chemical links to reduce toxic side effects [22,23]. It is obvious that the long-term effect of a chemical link is superior to that of a simple physical blend. QAS fragments are chemically bonded to the preferred macromolecules containing aromatic rings and a large number of hydroxyls [24,25,26].

In recent years, tannic acid (TA), a natural product extracted from plants and an excellent cross-linker with appropriate antimicrobial and antioxidant potential, has attracted great interest from food preservation researchers. Picchio et al. [27] prepared a packaging film by linking tannic acid and casein, and Halim et al. [28] reported membranes combined tannic acid with chitosan, gelatin, and methylcellulose for the preservation of fruits, the biopolymeric film treated with TA reduced the weight loss rate and browning index of fruits. However, there are few studies on the chemical grafting modification of tannic acid, especially the modification of tannic acid molecules by chemical reaction to improve the antibacterial properties of tannic acid. A recent study [29] reported the synthesis of a mono-quaternary TA by grafting QASs to enhance the leachability and biocompatibility of antibacterial medical thermoplastic polyurethane catheters. While this quaternary tannic acid exhibits strong antimicrobial properties, its poor water solubility and limited water-holding capacity may restrict its application in food packaging. For instance, packaging materials with poor water-holding capacity are unable to absorb water vapor on their surface, leading to internal moisture buildup that provides an ideal environment for rapid bacterial growth, ultimately causing food spoilage [26].

Herein, a water-soluble bi-quaternized tannin (BQTA), different from mono-quaternized tannins (QTA) reported in the literature [29], and contains more hydrophilic and antibacterial quaternary ammonium groups, was synthesized by a two-step reaction. The synthesized BQTA was then used to cross-link PVA. Various characterization techniques were applied to the obtained BQTA/PVA composite films, and physicochemical and biological characteristics of the composite films were assessed to obtain a biodegradable material for the packaging or enclosing of food to prevent putrefaction.

## 2. Materials and Methods

### 2.1. Materials

TA was purchased from Shanghai McLean Biochemical Co., Ltd. (Shanghai, China). Polyvinyl alcohol (PVA, average molecular weight: 118,000–124,000) was purchased from Sinopharm Chemical Reagent Co., Ltd. (Shanghai, China). 1-Bromododecane, 1,6-dibromohexane, and dimethylformamide (DMF) were purchased from Shanghai Adamas Reagent Co., Ltd. (Shanghai, China). *N*,*N*,*N*′,*N*′-tetramethyl-1,6-hexanediamine (TMHDA) and *N*,*N*-dimethyldodecylamine was purchased from Shanghai Haohong Biomedical Technology Co., Ltd. (Shanghai, China). N-hexane and sodium carbonate were purchased from Shanghai Aladdin Biochemical Technology Co., Ltd. (Shanghai, China). All chemicals were of analytical grade.

### 2.2. Synthesis of Br-BQAS and BQTA

**Figure 1:**
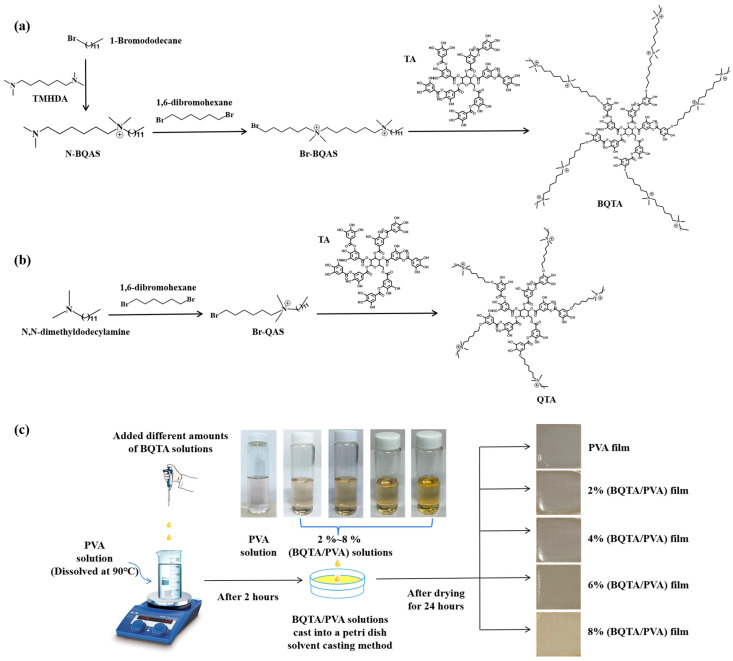
Synthesis of bisquaternary ammonium tannin (BQTA) (**a**) and quaternary ammonium tannin (QTA) [29] (**b**). Preparation process of BQTA/PVA composite membrane (**c**).

The experimental flow chart of the quaternization reaction of BQTA is shown in Figure 1a. First, TMHDA and 1-Bromododecane were blended in a molar ratio of 3:1 and reacted under magnetic stirring at 50 °C. After 70 h, the reaction was stopped, the solvent was evaporated and removed, and the entire solution was poured into excess n-hexane and stirred. The resulting substance was N-QAS. Then, 1,6-dibromohexane and N-QAS were mixed in ethanol at 5:1 and were stirred magnetically at 45 °C for 40 h. The ethanol solvent was removed by rotary distillation, and the remaining substance was washed with a large amount of n-hexane to remove the upper milky suspension and retain the orange solid precipitated at the bottom. The obtained precipitate was Br-BQAS, which was dried at room temperature for three days before storage and subsequent processing.

TA, sodium carbonate, and Br-BQAS were mixed into the three-neck flask according to the molar ratio of 1:20:20, and DMF was added as a solvent. The reaction was conducted at 80 °C for 20 h under a N_2_ atmosphere under condensation. After the reaction was stopped, the reaction liquid was slowly added to the excessive ethyl acetate solution in a vigorously agitated solution to precipitate the product. This process was repeated three times. The precipitates were dried in a vacuum-drying oven at 60 °C for 18 h. The brown solid powder obtained after drying was bisquaternary ammonium tannic acid and was labeled as BQTA. As a comparison, QTA reported in the literature was also synthesized, as shown in Figure 1b. Firstly, Br-QAS was synthesized, and then Br-QAS was grafted on tannic acid to synthesize QTA.

### 2.3. Preparation of BQTA/PVA Composite Films

BQTA/PVA composite films were prepared using the solution casting method. Firstly, 0.6 g PVA powders were dissolved in an appropriate amount of deionized water at 90 °C for 2 h. Then, the prepared BQTA was added to the PVA solution, and the mass ratio of BQTA to PVA was set to 2, 4, 6, and 8%, respectively. The obtained mixtures of BQTA and PVA were stirred continuously at 90 °C for 2 h to obtain the casting solution. BQTA/PVA composite film was obtained by pouring the casting solution on a glass plate with a diameter of 90 mm and then drying in a vacuum-drying box at 25 °C for 24 h. The preparation process of the BQTA/PVA composite membrane is shown in Figure 1c.

### 2.4. Characterization of BQTA

The hydrogen spectrum of BQTA was measured by ^1^H nuclear magnetic resonance (NMR) (AMX400M, Bruker, Billerica, MA, USA) at a frequency of 400 MHz and a scan number of 16 times. BQTA was dissolved in 600 mm^3^ deuterated water (D_2_O) at a concentration of 10 mg·cm^−3^. The infrared spectra of the prepared samples were scanned by an infrared spectrometer (FTIR Affinity-1, Shimadzu, Tokyo, Japan) in the wavenumber range of 400~4000 cm^−1^, and the BQTA solid powder and potassium bromide were directly taken and pressed at a ratio of 1:100. The zeta potential of BTQA was measured with a nanoparticle particle size meter (Zeta Sizer Nano ZS, Malvern, UK).

### 2.5. Antibacterial Properties

In this study, *Escherichia coli* (*E. coli*, ATCC 25922) was used as a Gram-negative bacteria, and *Staphylococcus aureus* (*S. aureus*, ATCC 25923) was used as a Gram-positive bacteria.

The filter paper diffusion method is as follows: TA, Br-QAS, QTA, Br-BQAS, and BQTA were each dissolved in DMSO (Dimethyl sulfoxide) to a concentration of 1 mg·cm^−3^ to prepare the test solutions. A 300 mm^3^ volume of bacterial solution at 10^6^ CFU·cm^−3^ was evenly spread over the surface of a solid culture dish. Sterilized filter papers, 6 mm in diameter, were then placed on the surface of the bacteria-inoculated medium. Subsequently, 20 mm^3^ of the prepared test solutions were added to the corresponding filter papers, and the culture dish was incubated at 37 °C for 12 h. The diameter of each inhibition zone was measured using a vernier caliper, with each measurement repeated 3 times and the average value taken. The antibacterial properties of each substance were then assessed by comparing the average sizes of the inhibition zones. For the BQTA/PVA composite membrane, the method differed in that the 6 mm BQTA/PVA composite membrane was directly applied to the surface of the inoculated solid medium for testing [30].

The plate counting method was used to adjust the concentration of antibacterial agent (TA, Br-QAS, QTA, Br-BQAS) and BQTA/PVA composite membrane to 10 μg·cm^−3^ and 350 μg·cm^−3^, respectively. The bacteria in the logarithmic growth phase were diluted to a concentration of 10^6^ CFU·cm^−3^, and 1 cm^3^ of the bacterial solution was dispersed in a 5 cm^3^ PBS solution, and then 1 cm^3^ of the sample solution was added. The blank control without an antibacterial agent was set up and co-cultured for about 3 h in an incubator at 37 °C. Subsequently, 300 mm^3^ mixed bacterial solution was evenly coated on the surface of the culture dish and finally cultured at 37 °C for 12 h, then the number of colony-forming units (CFU) was counted [31]. The antibacterial performance was calculated as follows:

(1) $$Antibacterial\;ratio=\frac{{CFU}_{0}-{CFU}_{test}}{{CFU}_{0}}\times 100\%$$

where *CFU*_0_ and *CFU_test_* correspond to tests without and with antibacterial agents.

### 2.6. Antioxidant Properties

The antioxidant properties of the samples were evaluated by the scavenging ability of DPPH (2,2-diphenyl-1-picrylhydrazyl) [32]. First, 75 μmol·dm^−3^ DPPH solution was prepared. Next, 1 cm^3^ of different concentrations of BQTA were added to the 4 cm^3^ DPPH working solution and stood in the dark for 2 h to ensure that the free radical scavenging reaction was fully carried out. Finally, the absorbance of the sample at 517 nm was measured by ultraviolet spectrophotometer (Cary 5000 UV-Vis-NIR, Agilent Technology, Beijing, China), and the scavenging activity of the sample on DPPH free radicals was calculated. The antioxidant performance was calculated as follows:

(2) $$DPPH\;radical\;scavenging\;activity=\left(1-\frac{A_{sample}}{A_{blank}}\right)\times 100\%$$

where *A_sample_* and *A_blank_* correspond to absorbance tests without and with antibacterial agents.

### 2.7. Cytotoxicity

CCK-8 method was used to explore the toxic effects of antibacterial agents or films on NIH3T3 cells [33]. First, DMEM liquid medium containing 10% fetal bovine serum was prepared, and the cells were incubated. The antibacterial agent or films were prepared according to the concentrations of 10 μg·cm^−3^, 100 μg·cm^−3^, and 1000 μg·cm^−3^. Then, different concentrations of antibacterial agents or films were fully contacted with the cells and co-cultured, and then CCK-8 reagent was added to continue the culture. Finally, the absorbance at 450 nm was detected by a microplate reader (SPARK 10M, TECAN, Männedorf, Switzerland) to calculate cell viability.

### 2.8. Morphology Characterization

The surface morphology of the films was observed by a field emission scanning electron microscope (SEM) (S-4800, HITACHI, Tokyo, Japan). The samples were broken with liquid nitrogen for cross-section observation.

### 2.9. Mechanical Properties

The tensile strength was measured by a tension machine (Shimadzu AG-X plus 10KN electronic universal testing machine, Tokyo, Japan) with films cut into dumbbell-shaped specimens, with a length and a width of 25 mm and 4 mm at the narrow section, respectively. The experiment was conducted at a stretching speed of 50 mm/min, with five samples of each film type prepared for repeated measurements. The average value was calculated from the test results.

### 2.10. Structural Characterization of Films

The crystal structure of the composite films was directly analyzed by an X-ray powder diffractometer (D8 Advance Bruker, Karlsruhe, Germany). In this process, CuKa was used as the target pole, the scanning speed was 10°/min, and the scanning range was 10~80°. The crystallinity was calculated using MDI Jade 6 software.

The infrared characterization of the composite films can be conducted following the method used for BQTA infrared characterization. The primary difference is that the film must first be ground into a powder using a file, after which the powder is mixed with potassium bromide and pressed into a pellet.

### 2.11. Thermal Properties

The thermal behavior was performed by a thermogravimetric analyzer (TA, NETZSCH, Hanau, Germany) under a nitrogen atmosphere from 25 °C to 600 °C at a heating rate of 10 °C·min^−1^.

### 2.12. Optical Performance

The transmittance UV–Vis spectrum of composite films was tested on an ultraviolet spectrophotometer. The transmission wavelength of the prepared films ranged from 300 nm to 800 nm.

### 2.13. Water Contact Angle

The contact angle between the composite film surface and water was measured using a video optical contact angle meter (DSA100, KRUSS, Hamburg, Germany). A sample of the composite film was placed on a movable stage, and 3 mm^3^ of distilled water was dispensed from a micro-syringe at the needle’s outlet. The stage was then slowly raised until the film made contact with the water droplet. At this precise moment, images of the droplets were captured using a lens and light source, and the droplet shapes were analyzed to determine the static contact angle. The contact angle data for each sample were subsequently recorded.

### 2.14. Anti-Fogging Performance

By covering the packaging box coated with BQTA/PVA coating on the cup mouth containing 95 °C water for observation, the above phenomena were recorded by the camera.

### 2.15. Preservation Application

The preservation efficacy of the casting solution was assessed over a 7-day period under controlled conditions of 25 °C room temperature and 50% relative humidity. Fresh, uniformly sized, and evenly ripe strawberries were carefully selected to ensure consistent results. Each strawberry was thoroughly washed with distilled water, air-dried, and then immersed in the BQTA/PVA casting solution for exactly 30 s. After immersion, the strawberries were placed on sterile Petri dishes. The ripeness and decay of the strawberries were systematically monitored on the 1st, 3rd, 5th, and 7th days, with observations meticulously documented through photography.

## 3. Results

### 3.1. Structural of BQTA

**Figure 2:**
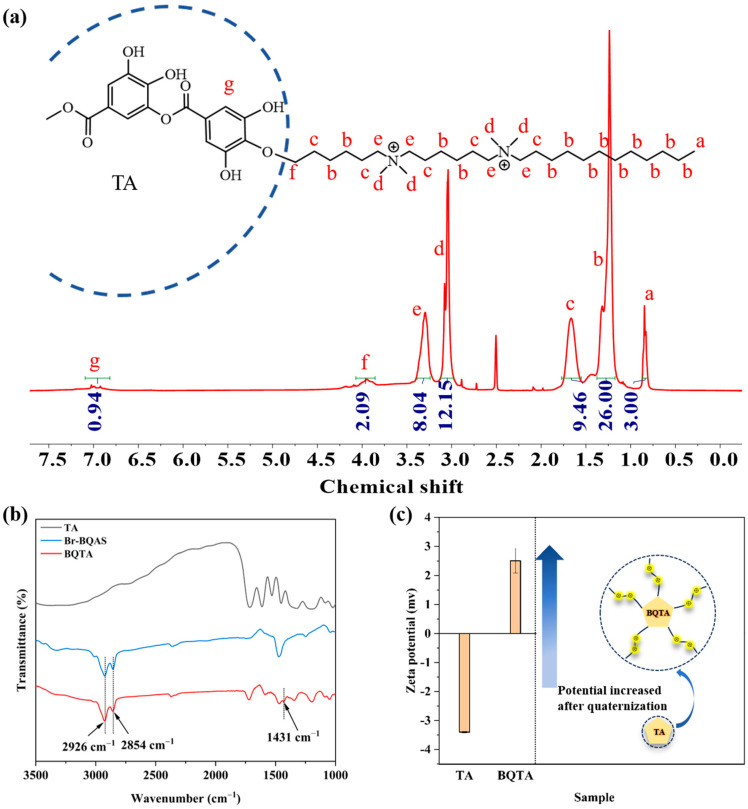
^1^H NMR spectra of the synthetic BQTA (**a**) FTIR spectra of the TA and BQTA (**b**). The zeta-potentials of TA and BQTA (**c**).

BQTA was synthesized through two steps reaction. Initially, a bisquaternary ammonium bromide compound (Br-BQAS) was synthesized. Subsequently, Br-BQAS was grafted onto TA to prepare BQTA. The chemical structures of BQTA were characterized via ^1^H NMR and FTIR. As shown in Figure 2a of the BQTA nuclear magnetic resonance hydrogen spectrum, based on the hydrogen atom on the methyl group at one end of the side chain of the substance, the peak area is set to 3, corresponding to the three hydrogen atoms on the methyl group. The peak areas of a, b, c, d, e, f, and g on the BQTA nuclear magnetic resonance spectrum correspond to the ratio of the number of hydrogen atoms at a, b, c, d, e, f, and g on the BQTA to 3:26:8:12:8:2:1, respectively, which confirms the successful preparation of BQTA, the contrastively experimental results show, in according to the spectrum of QTA, the number ratio of protons is approximately 3:28:10:2:1 [29].

In addition, the FTIR characterizations of TA and BQTA were conducted, and the results are shown in Figure 2b. BQTA has an absorption peak at 1431 cm^−1^, which is the stretching vibration peak of the C-N bond, and two absorption peaks are found at wavenumbers of 2854 cm^−1^ and 2926 cm^−1^, corresponding to the stretching vibration peaks of methyl (-CH_3_-) and methylene (-CH_2_-), respectively, while TA has no absorption peak at the above wave number, indicating that there is a long carbon chain structure in BQTA. Further, the zeta-potentials of natural TA and synthetic BQTA in Figure 2c show the wide difference in zeta potential from negative to positive. The above data indicate the successful synthesis of BQTA.

The oxygen atoms of all the phenolic hydroxyl groups in TA could potentially react with the intermediate product of Br-BQAS. According to the method described in the literature [29], the grafting ratio of the synthetic BQTA was determined to be 63.0%.

### 3.2. Antibacterial Activity of BQTA

**Figure 3:**
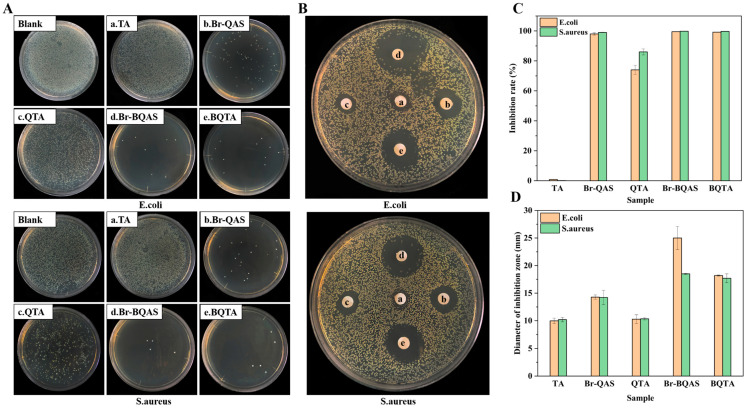
Antibacterial activity of BQTA. Colony diagram (**A**), inhibition zone diagram (**B**), inhibition rate (**C**), and inhibition zone size (**D**) of PVA films with different BQTA content (a—antibacterial effects of TA, b—Br-QAS, c—QTA, d—Br-BQAS, e—BQTA).

Antibacterial properties of BQTA were explored, and for comparison, that of TA, QTA, and their respective intermediates were measured simultaneously. The experimental results are shown in Figure 3. From the results of the plate counting method in Figure 3A,C, it can be seen that there were no bacteria on the surfaces of the Petri dishes coated with BQTA specimen at 10 μg·cm^−3^ of the test concentration, the antibacterial rates of BQTA (e) have all reached 100%, which is significantly greater than 74.1% and 86.3% of QTA (c) against *E. coli* and *S. aureus*, respectively. The results of the filter paper diffusion method in Figure 3B,D showed that when all samples were at a concentration of 1 mg·cm^−3^, the inhibition zone diameters of BQTA against *E. coli* and *S. aureus* were 25.0 mm and 18.5 mm, respectively, QTA was 18.2 mm and 17.7 mm. The inhibition zone of BQTA is larger than that of QTA, which further illustrates that BQTA has better antibacterial properties than QTA reported [29].

### 3.3. Antioxidant Activity of BQTA

**Figure 4:**
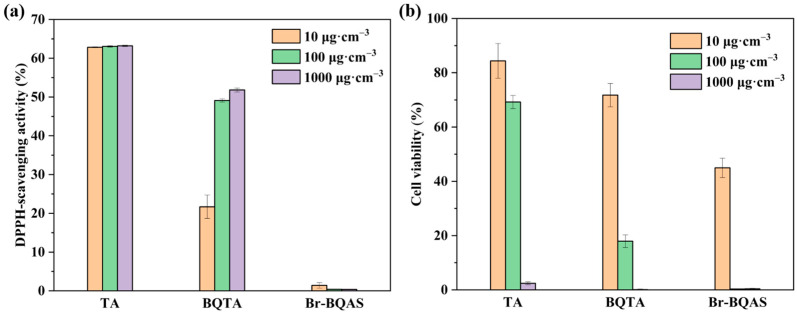
Antioxidant performance (**a**) and cell viability results (**b**) of TA, BQTA, and Br-BQAS.

TA has been known to be an excellent antioxidant due to multiple phenolic hydroxyl groups, which can help delay the oxidation of foods [25]. Partial phenolic hydroxyl groups on BQTA were linked with quaternary ammonium chains, and it will inevitably affect its antioxidant properties. DPPH, a cell-permeable, stable free radical, is used to evaluate the antioxidant activity of samples as free radical scavengers or hydrogen donors. Figure 4a shows the DPPH free radical scavenging activity of TA, BQTA, and some associated samples.

As expected, TA maintained a free radical scavenging rate of more than 60% at 10~1000 μg·cm^−3^, while the corresponding observed values of BQTA were about 22~52%. The data demonstrate that, although partial substitutions of hydroxyl groups reduce BQTA antioxidant activity, BQTA still has certain antioxidant activity for the presence of the remaining phenolic hydroxyl groups.

### 3.4. Cytotoxicity of BQTA

The biocompatibility of food packaging is directly related to human health and safety. In order to analyze the cytotoxicity caused by the presence of BQTA, NIH3T3 cells were used to detect the effects of BQTA composite by CCK-8 test. The results are described in Figure 4b. The substrate TA had a higher cell survival rate at a concentration of 100 μg·cm^−3^, and the cell survival rate of the precursor Br-BQAS was the lowest. The biocompatibility of the modified BQTA was greatly improved compared with the precursor Br-BQAS, which is also significant in the synthesis of the antibacterial agent.

### 3.5. Morphology and Mechanical Properties of the BQTA/PVA Composite Films

**Figure 5:**
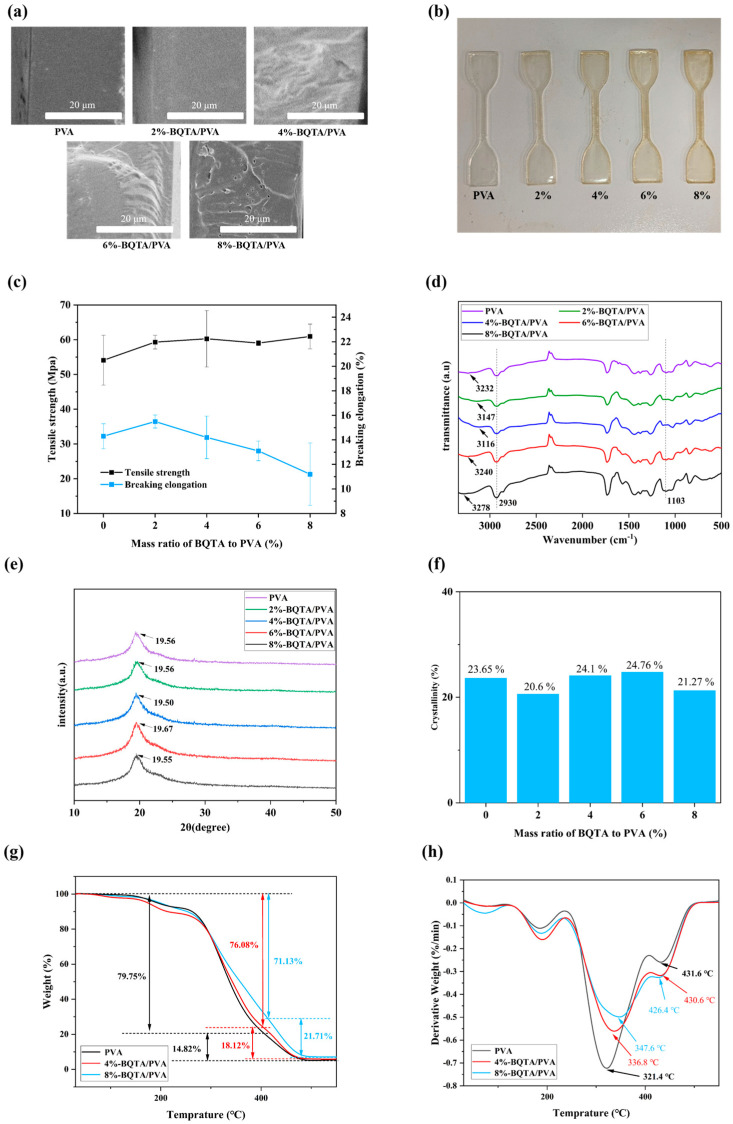
SEM images of the films cross-section (**a**). Photographic images (**b**) and experimental results (**c**) of the tensile tests. Structural characterizations of the films: FTIR spectrum (**d**), XRD patterns (**e**), and crystallinity results (**f**). Thermal properties of the films: TG curve (**g**), DTG curve (**h**).

The cross-section of the PVA film shown in Figure 5a is smooth and uniform, without voids and pores. When a small amount of BQTA was added, the cross-section of the composite film showed a compact structure, and with the amount of BQTA added increased, some micropores appeared, then gradually turned to larger pores. It was also observed that there was an increase in the viscosity of the casting solution, and the bubbles generated in the solution were difficult to dissipate.

These structural changes directly impact the mechanical properties of the composite films, which are critical for food packaging materials to withstand external physical stress during storage and transportation. Figure 5b shows the original samples that will be tested at a stretching speed of 50 mm/min. According to Figure 5c, both tensile strength and elongation of the composite samples loaded with 2% BQTA are obviously improved in comparison with original PVA samples; however, as the content of BQTA further increased, the elongation of the composite film deteriorated, and there was a slight increase in tensile strength. SEM results validate this observation, showing that the excessive addition of BQTA leads to void formation. These pores cause uneven stress distribution during tensile testing, ultimately resulting in premature film fracture. Thus, a small amount of BQTA existing in the biopolymer-based packaging materials assisted the enhanced plasticity and elasticity properties.

### 3.6. Structural Characterization of the BQTA/PVA Composite Films

To investigate the internal structure of the composite films, FTIR (Figure 5d) and XRD (Figure 5e,f) analyses were conducted following the observed changes in their mechanical properties. Firstly, all materials displayed prominent peaks at 2930 cm^−1^ and 1103 cm^−1^, corresponding to the stretching vibrations of the methylene group and the C-O bond, respectively. These peaks are characteristic of both PVA and BQTA. The pure PVA film showed a characteristic O-H stretching peak at 3232 cm^−1^. However, when 2% to 8% BQTA was added, this peak shifted, with new peaks appearing at 3147, 3116, 3240, and 3278 cm^−1^. This shift likely occurs due to hydrogen bond formation between the PVA and BQTA, altering the O-H bond length and energy, which changes the vibration frequency and wavenumber [34]. The hydrogen bonding between the polymers strengthens their interaction, explaining the increased viscosity of the BQTA/PVA casting solution with more BQTA. This increase in viscosity subsequently leads to the formation of micropores in the dried composite film.

The mechanical properties of polymer materials are also significantly influenced by the degree of polymer crystallinity [35]. At a high degree of crystallinity, polymer materials become harder and more thermally stable but are also accompanied by much more fragile features, whereas amorphous polymer structures are more elastic and possess an enhanced impact resistance [36]. X-ray diffractograms of pure PVA and BQTA-reinforced PVA samples are shown in Figure 5e. In all the samples for various proportions, the crystalline peaks are observed between 19.50° and 19.67°, and the variations of corresponding crystallinity calculated by Jade can be seen in Figure 5f. There was a decrease in crystallinity for 2% BQTA/PVA composite and increases for other composites, which is consistent with the above mechanical results of an enhanced elongation at break for 2% BQTA/PVA composite and the decreases for the other composites. This is due to the crystalline degree in PVA is determined by the number of PVA chains packing together and BQTA breaks the orderly arrangement of the PVA polymer chains, and more BQTA addition, and crystallization was flow-induced at high polymer viscosity.

This also reasonably explains why the tensile strength of the composite film increases and the elongation at break decreases in the mechanical properties analysis of the composite film.

### 3.7. Thermal Properties of the Films

The maximum decomposition temperature (*T_max_*) and weight loss (%) for each thermal decomposition step of PVA and BQTA/PVA composite films were obtained from TGA (Figure 5g) and differential TGA (DTG) (Figure 5h) curves.

A slight weight loss at the initial stage is found below 100 °C for every specimen due to the absorbed free water volatilization. The second thermal stage refers to dehydration bound mainly in hydroxyl groups of the sample around 230 °C, and the third thermal stage can be attributed to the decomposition of the thermal degradation of polymer molecules. The decomposition of the polyvinyl alcohol (PVA) side chains occurs at *T_max_* ≈ 321.4 °C, with a total weight loss of 79.75%. The decomposition of the PVA main chain occurs at *T_max_* ≈ 431.6 °C (weight loss of 14.82%). For PVA composites containing 4% and 8% BQTA, side chain degradation occurs at *T_max_* ≈ 336.8 °C and 347.6 °C, with corresponding weight losses of 76.08% and 71.13%. The main chain decomposition of PVA occurs at *T_max_* ≈ 430.6 °C and 426.4 °C, with a weight loss of 18.12% and 21.71%. These data indicate that the addition of BQTA enhances the thermal stability of PVA side chains, likely due to hydrogen bonding between the phenolic hydroxyl groups in BQTA and the alcoholic hydroxyl groups in PVA, which improves the thermal stability of PVA.

### 3.8. UV Shielding Analysis of BQTA/PVA Composite Films

**Figure 6:**
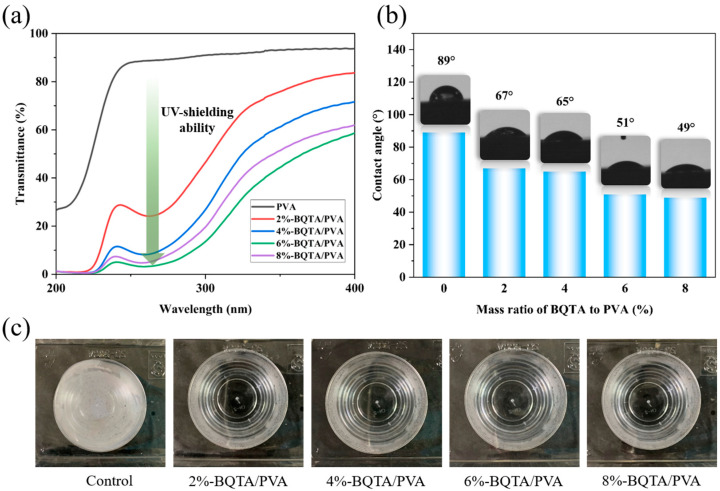
UV–Vis spectra (**a**), water contact angle (**b**), and anti-fog performance test (**c**) of composite films.

UV irradiation is one of the key factors affecting food spoilage and nutrient loss. The UV shielding was carried out by UV–Vis spectrophotometer, and the resulting transmittances are presented in Figure 6a. As shown, the transmittance of the composite film in the near ultraviolet region is much lower than that of the pure PVA film, indicating that the BQTA composite film has a better ultraviolet barrier function, which can reduce the degree of ultraviolet irradiation of food and prolong the shelf life of food. This effect is attributed to the multiple aromatic rings present in the structure of tannic acid. These aromatic rings can absorb UV light as the UV energy interacts with the π-electrons within the rings, inducing electronic transitions [37].

### 3.9. Anti-Fogging Performance of BQTA/PVA Composite Films

Anti-fogging performance of the packaging material can enhance the display of packaged food in high-humidity environments without impairing the sealing performance of the packaging material, which is a critical factor in preserving food quality and consumers’ acceptance.

BQTA/PVA composite was coated on the inner surface of a plastic box to evaluate the anti-fogging performance. Figure 6c elucidates that all the plastic boxes coated with the composite coating have high transparency, except for the control, and Figure 6b presents a detailed visualization of the water contact angles of composite films with different BQTA contents. With the increase in BQTA content, the surface of the composite film is more hydrophilic. This performance can prevent water formed inside the packaging due to transpiration during the transportation of fruits and vegetables, thereby avoiding a favorable environment for bacterial growth and preventing food spoilage [26].

### 3.10. Antibacterial Activity of Composite Films

**Figure 7:**
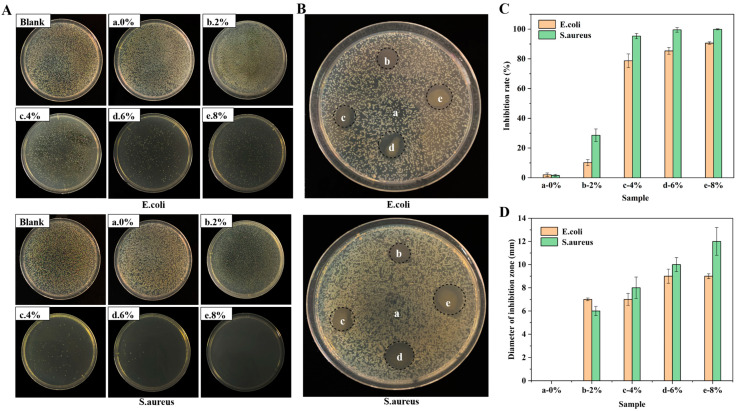
Antibacterial activity of BQTA/PVA composite films. Colony diagram (**A**), inhibition zone diagram (**B**), inhibition rate (**C**), and inhibition zone size (**D**) of PVA films with different BQTA content (a—0%, b—2%, c—4%, d—6%, e—8%).

Used as an antibacterial additive in food packaging material, BQTA was dispersed in casting PVA solution, and the antibacterial effects are shown in Figure 7. It can be observed that there are a large number of colonies in the blank control group (blank) and pure PVA group (a). When the content of BQTA in the film was more than 4%, the number of colonies of *E. coli* and *S. aureus* began to become scarce. When the content of BQTA was more than 6%, the inhibition rate of the composite films on *E. coli* reached over 85%, and the rates of *S. aureus* were 99.5%. Figure 7B,D also show that the inhibition zone of the different composite films increases from 6 to 12 mm, while the pure PVA film placed in the center has no obvious inhibition zone. The above results show that the addition of BQTA to the matrix greatly improves bactericidal efficiency.

### 3.11. Antioxidant Activity of Composite Films

**Figure 8:**
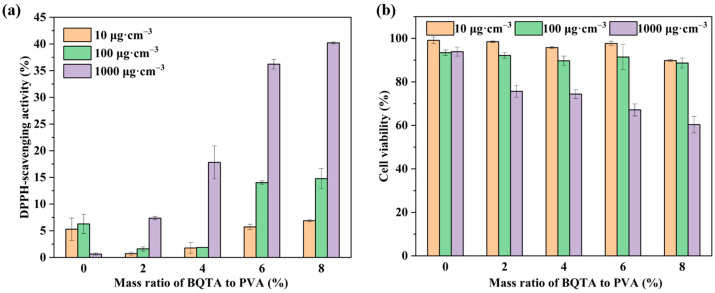
Antioxidant performance (**a**) and cell viability results (**b**) of composite films.

The antioxidant activity of films is an important parameter in food packaging applications. Further antioxidant characterizations of BQTA were performed with the incorporation of a PVA matrix. Figure 8a demonstrates the effects of small BQTA (0~8%) on the antioxidant properties of PVA composite films. It found that the scavenging effect of the composite films on radicals gradually enhanced as the content of BQTA increased, and at a concentration of 1000 μg·cm^−3^, the free radical clearance reached 40.2%. In contrast to the pure PVA, obviously, the composite film with small BQTA showed excellent antioxidant activity.

### 3.12. Cytotoxicity of Composite Films

As shown in Figure 8b, the addition of the antibacterial agent BQTA reduced the cell viability of the composite film. However, when the film was at a concentration of 100 μg·cm^−3^, the cell viability remained above 85%, which is still within an acceptable range. These results indicate that the coating is a packaging material suitable for food preservation.

### 3.13. Application to Strawberry Preservation

The dual function of antioxidation and antibiosis shows that BQTA has great potential to be applied as a smart and active packaging, which would play an important role in ensuring food safety and improving food storage quality.

**Figure 9:**
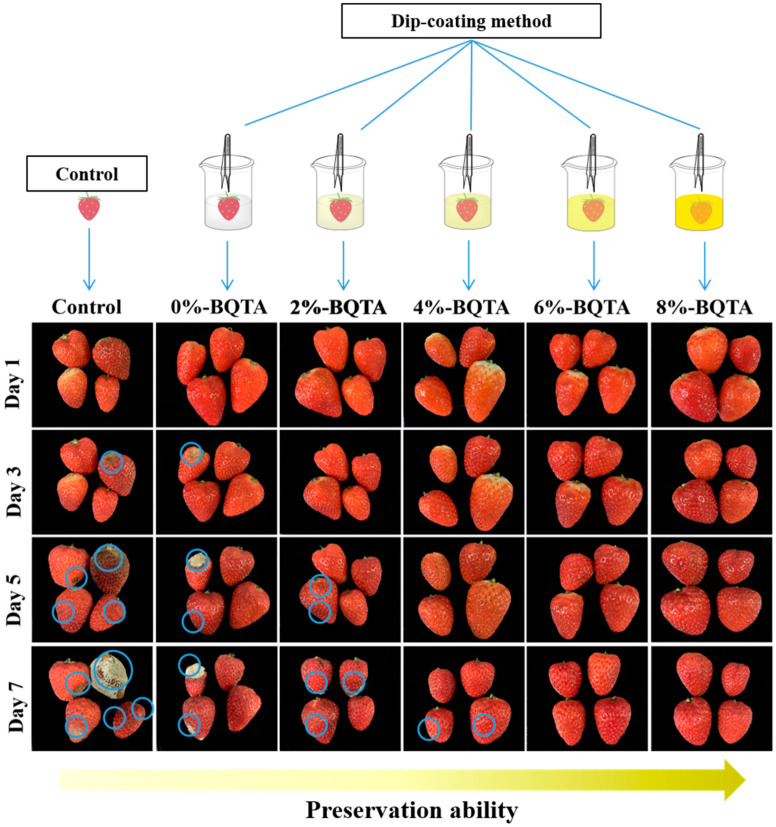
Appearance changes of strawberries stored at 25 °C.

Strawberries were selected to evaluate the potential in active food packaging because strawberries are more perishable during storage after harvest. As shown in Figure 9, strawberries in the control group and the pure PVA group decayed after 3 days of storage (marked with blue circles), indicating that PVA has no inhibitory effect on the microbes. In contrast, the experimental groups with PVA-BQTA maintained their original color and showed no sign of decay until the seventh day. Hence, the PVA-BQTA could effectively prolong the shelf life of strawberries, demonstrating its potential in active food packaging.

## 4. Conclusions

In summary, a BQTA was first synthesized by grafting a synthetic Br-BQAS onto TA. Then, the PVA multifunctional composite film was successfully loaded with BQTA. The prepared BQTA compound processes both residual phenolic hydroxyl groups and surface positive charge properties, thus displaying excellent dual function of antioxidation and antibiosis. The designed BQTA/PVA multifunctional composite film exhibits excellent anti-ultraviolet, anti-fog, and antibacterial properties, which can effectively delay the deterioration of fruits and vegetables. In addition, the biocompatibility, thermal stability, renewable origin, and biodegradation confirm the potential qualification of BQTA-reinforced polymer-based packaging materials. These findings provide new strategies for the innovative development of packaging materials.

## 5. Patents

Laiqi Li, Sufang Li, and Yangyi Gong. A dicationic tannic acid and its preparation method and application. Publication patent number (Patent granted), CN115677799B.

## Data Availability

The raw data supporting the conclusions of this article will be made available by the authors upon request.
